# Identifying hub circadian rhythm biomarkers and immune cell infiltration in rheumatoid arthritis

**DOI:** 10.3389/fimmu.2022.1004883

**Published:** 2022-09-27

**Authors:** Pengfei Wen, Tao Ma, Binfei Zhang, Linjie Hao, Yakang Wang, Jianbin Guo, Wei Song, Jun Wang, Yumin Zhang

**Affiliations:** Department of Joint Surgery, Honghui Hospital, Xi’an Jiaotong University, Xi'an, China

**Keywords:** rheumatoid arthritis, synovial tissue, circadian rhythm, immune cell infiltration, bioinformatics analysis, biomarker

## Abstract

**Background:**

Rheumatoid arthritis (RA) is a chronic systemic autoimmune disease with symptoms characterized by typical circadian rhythmic changes. This study aimed to identify the hub circadian rhythm genes (CRGs) in RA and explore their association with immune cell infiltration and pathogenesis of RA.

**Methods:**

The differentially expressed CRGs (DECRGs) between RA and normal control samples were screened from Datasets GSE12021 and GSE55235. Gene Ontology, Kyoto Encyclopedia of Genes and Genomes, and Gene Set Enrichment Analysis were used to explore the potential functional mechanisms of DECRGs in RA. Weighted Gene Co-expression Network Analysis and Least Absolute Shrinkage and Selection Operator regression analysis were performed to identify hub CRGs of RA. CIBERSORT was conducted to compare the infiltration level of immune cells in RA and control synovial tissue and their relationship with hub genes. In addition, the diagnostic value of hub biomarkers was evaluated by the area under the receiver operator characteristic curve. Further, a nomogram prediction model was constructed and its significance for clinical decision-making was evaluated.

**Results:**

The green module was identified as the hub module associated with RA. Four hub CRGs (EGR1, FOSL2, GADD45B, and NFIL3) were identified and showed that they had the highest specificity and sensitivity for RA diagnosis, respectively. The expression levels and diagnostic values of these genes were externally validated in the dataset GSE55457. A nomogram prediction model based on the four hub CRGs was constructed and proved to have a certain clinical decision value. Additionally, the correlation analysis of immune cells with hub genes showed that all hub genes were significantly positively correlated with activated mast cells, resting memory CD4+ T cells, and monocytes. Whereas, all hub genes were negatively correlated with plasma cells, CD8+ T cells, and activated memory CD4+ T cells. Meanwhile, FOSL2 and GADD45B were negatively correlated with Tfh cells.

**Conclusion:**

Four hub CRGs were identified and showed excellent diagnostic value for RA. These genes may be involved in the pathological process of RA by disrupting the rhythmic oscillations of cytokines through immune-related pathways and could be considered molecular targets for future chronotherapy against RA.

## Introduction

Rheumatoid arthritis (RA) is a chronic systemic autoimmune disease. Most epidemiological studies in developed countries have shown that the prevalence of RA in adults is between 0.5 and 1% (1). The main pathology of RA is chronic synovitis of the joints, characterized by persistent synovial inflammation, synovial hyperplasia, and pannus formation, which could destroy bone and cartilage tissue and gradually lead to functional impairment of the joints (2, 3), as well as to serious complications such as cardiovascular disease (4, 5). However, a consensus on the pathogenesis of RA has long been lacking. It is well known that patients with RA have typical circadian rhythm clinical symptoms, such as significant joint stiffness and swelling in the morning. Mounting evidences have demonstrated that the development and treatment of RA are strongly associated with circadian rhythm (6–8). Therefore, an in-depth understanding of the molecular mechanisms underlying the circadian rhythm changes in the onset of RA is important for the early diagnosis of RA and the search for new therapeutic targets.

Immune factors play a crucial role in the overall pathogenesis of RA, although it is the result of a complex combination of genetic, environmental, and immune factors (9). Circadian rhythms acted by central or peripheral cellular clock genes can regulate endocrine, energy metabolism, and immune function related pathological conditions that are associated with several diseases such as cancer, neurodegenerative diseases, cardiovascular diseases, and RA (6, 10, 11). It has been reported that circadian rhythm disturbances due to shift work are closely related to the development of RA (7). This chronobiological factor is partly attributed, at least, to the underlying circadian rhythm patterns of cytokines and hormones (12, 13). The rhythmic changes of symptoms in RA patients have been shown to be accompanied by circadian oscillations in pro-inflammatory cytokine concentrations, where the levels of key inflammatory cytokines in RA (e.g., IL-6, TNF-α, and IL-1β) are significantly elevated at night (6, 14). Most immune cells express circadian rhythm genes (CRGs), and this rhythm has a major impact on immune cell function, including the synthesis and release of cytokines and chemokines at night, as well as regulating the function of the circadian immune system through pattern recognition receptors (15, 16). Additionally, in the chronic inflammatory conditions of RA, the production of glucocorticoids (GCs) as potent anti-inflammatory substances is insufficient or low at night, which leads to the exacerbation of morning symptoms in RA (17, 18). Altogether, the cumulative damage caused by circadian rhythm-mediated release of inflammatory cytokines, infiltration of immune cells, and disturbance of hormone levels may play a critical role in pathogenesis of RA.

With the development and widespread use of gene chips and high-throughput sequencing technologies, bioinformatics analysis can be used to identify novel genes and biomarkers for many diseases, including autoimmune diseases (19, 20). To identify circadian rhythm-related biomarkers in RA patients and to explore their possible molecular mechanisms to facilitate the treatment of RA. We screened the differentially expressed circadian rhythm genes (DECRGs) between RA and control samples from the Gene Expression Omnibus (GEO) database. Based on the screening of DECRGs, hub biomarkers were identified by a combination of weighted gene co-expression network analysis (WGCNA) and least absolute shrinkage and selection operator (LASSO) regression analysis. Subsequently, the infiltration level of immune cells in RA synovial tissue and the relationship between immune cells and hub genes were further analyzed, which provides a new perspective for the diagnosis and treatment of RA.

## Materials and methods

### Data collection and processing

**Figure 1:**
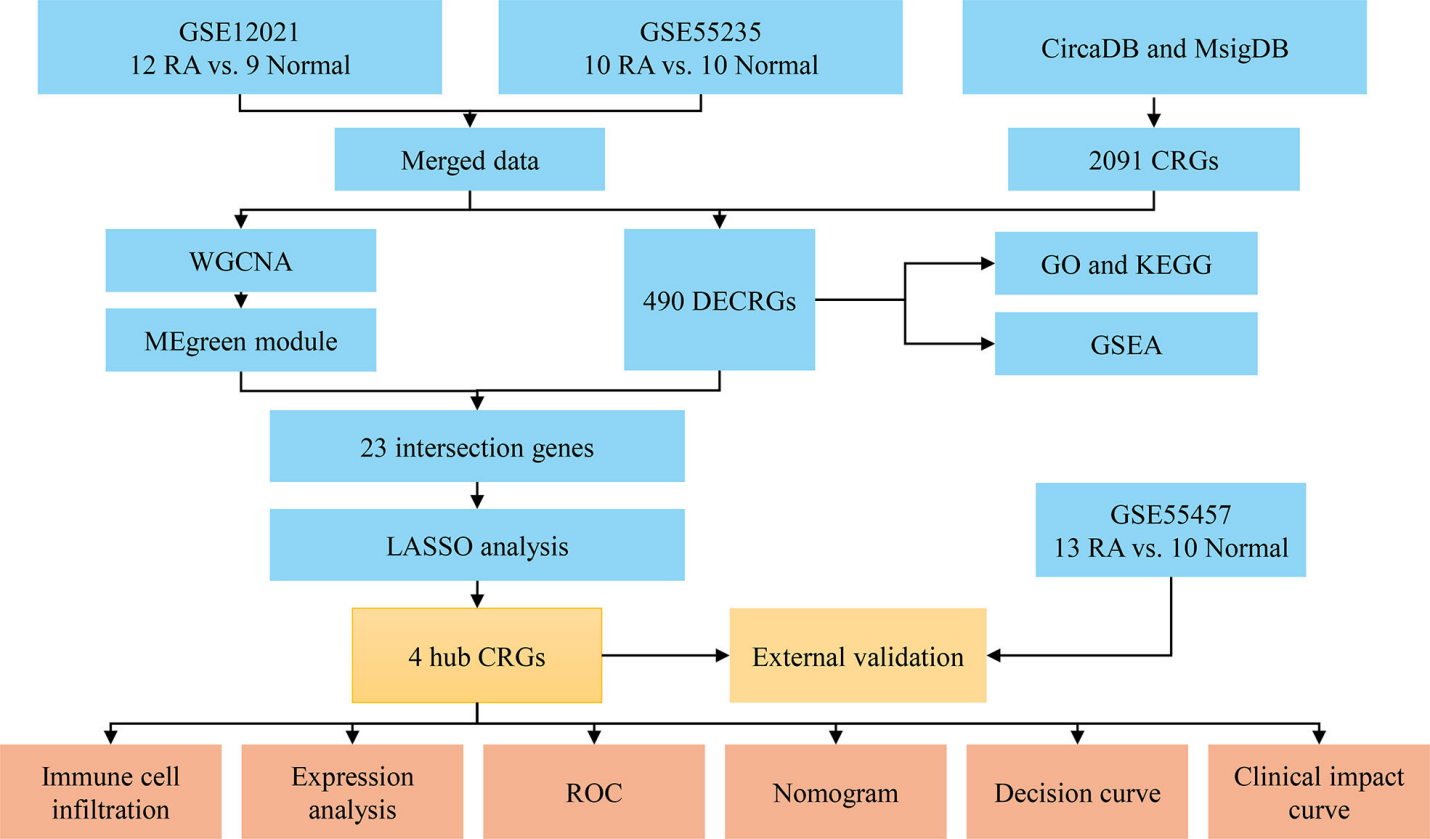
Flowchart of the analysis process. RA, rheumatoid arthritis; CRGs, circadian rhythm genes; DECRGs, differentially expressed circadian rhythm genes; WGCNA, weighted gene co-expression network analysis; GO, gene ontology; KEGG, kyoto encyclopedia of genes and genomes; GSEA, gene set enrichment analysis; LASSO, least absolute shrinkage and selection operator; ROC, receiver operating characteristic.

The workflow of this study was illustrated in **Figure 1**. Three RA datasets (GSE12021, GSE55235, and GSE55457) were downloaded from the GEO database (https://www.ncbi.nlm.nih.gov/geo/). The dataset GSE12021 consisted of 12 RA samples and nine normal samples; GSE55235 contained 10 RA samples and 10 normal samples; GSE55457 included 13 RA and 10 normal samples. The R package ‘sva’ was used to perform batch correction on all datasets and merged the GSE12021 and GSE55235 datasets. In addition, 2,091 CRGs were collected from the databases CircaDB (http://circadb.org) and MsigDB (https://www.gsea-msigdb.org/gsea/msigdb) (**Supplementary Table 1**).

### Screening of DECRGs and enrichment analysis

The expression data of 2,091 CRGs were extracted from the datasets GSE12021 and GSE55235. Then, 490 DECRGs between RA and normal control groups were obtained by differential analysis of the data using the ‘limma’ R package. The heatmap and volcano map were plotted using the R package ‘pheatmap’ and ‘ggplot2’, respectively. The screening criteria for DECRGs were adjusted p-value < 0.05 and |log fold change (FC)| > 1. To understand the functions of these DECRGs, Gene Ontology (GO) analysis and Kyoto Encyclopedia of Genes and Genomes (KEGG) pathway analysis were performed by the R package ‘clusterProfiler’, with p and q values < 0.05 being a significant difference. Meanwhile, Gene Set Enrichment Analysis (GSEA) was performed for the RA and normal groups, and the top five gene sets with active enrichment were shown.

### Identification of hub CRGs

Weighted gene co-expression network analysis (WGCNA) was performed for the top 25% of genes in the merged dataset. A soft threshold (β) was selected and validated by the ‘PickSoftTreshold’ function of WGCNA (20). Genes with the highest inter-module connectivity were selected as candidate hub genes. Genes with biological significance usually have a high Gene significance (GS). Candidate hub genes associated with RA were screened based on the criteria of module membership (MM) > 0.8 and GS > 0.5. The intersection of the candidate hub genes with DECRGs was taken, and further Least Absolute Shrinkage and Selection Operator (LASSO) regression analysis was performed on the intersecting genes using the R package ‘glmnet’ to identify the final hub CRGs for RA.

### Immune cell infiltration analysis

CIBERSORT was used to evaluate the immune cell infiltration in the synovial tissue of each sample in the datasets GSE12021 and GSE55235. Histograms were plotted to show the proportion of immune cells in RA versus normal samples. Violin plots (R package ‘vioplot’) were drawn to reveal the differences in expression levels of 22 immune infiltrating cells. Subsequently, the relationships between immune cells and each hub CRG were further explored by Spearman’s correlation analysis and presented as lollipop plots.

### Diagnostic efficiency of hub CRGs

To evaluate the diagnostic value of the screened hub CRGs for RA, a differential analysis was performed for each hub gene, and the receiver operating characteristic (ROC) curve of each hub gene was performed with the R package ‘pROC’. Meanwhile, the R package ‘rmda’ was used to generate the nomogram, calibration curve, decision curve, and clinical impact curve of hub genes.

### Validation of hub CRGs

The expression levels and diagnostic values of hub CRGs were validated using a separate external dataset GSE55457. The validation results were presented as violin plots and ROC curves, respectively.

## Results

### Identification and enrichment analysis of DECRGs

**Figure 2:**
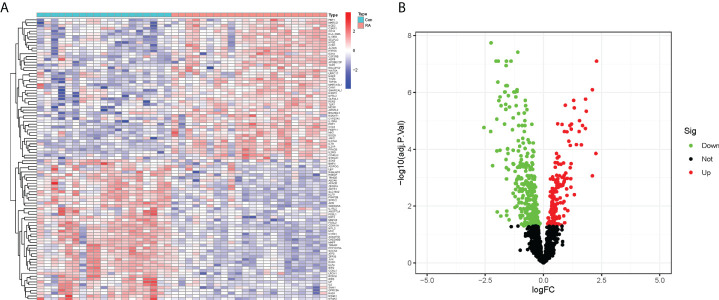
Identification of DECRGs between RA and control groups. **(A)** Heatmap of expression differences of CRGs (Green: low expression level; Red: high expression level). **(B)** Volcano plot of DECRGs (Green: down-regulated genes; Red: up-regulated genes).

In this study, the expression of 2091 CRGs was extracted from the datasets GSE12021 and GSE55235, and the expression differences of these genes were compared between the RA and control groups (**Figure 2A**). Further, a total of 490 DECRGs were obtained (**Supplementary Table 2**), of which 333 genes were down-regulated and 157 genes were up-regulated as presented in the volcano plot (**Figure 2B**).

**Figure 3:**
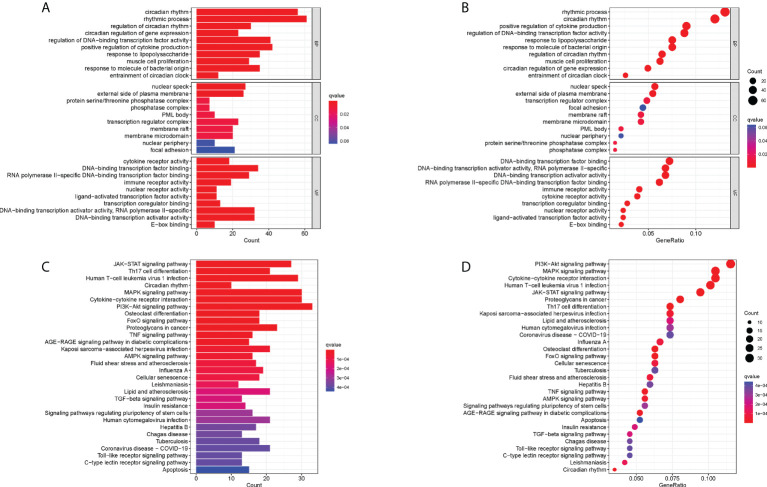
Functional correlation analysis of DECRGs between RA and control groups. **(A, B)** The results of GO analysis were presented by bar plot and bubble graph. **(C, D)** The results of KEGG pathway analysis were shown by bar plot and bubble graph. BP, biological process; CC, cellular component, MF, Molecular function.

GO enrichment analysis was performed to understand the biological functions of these RA-related DECRGs. In terms of biological processes, the results revealed that DECRGs were mainly enriched in circadian rhythm, rhythmic process, regulation of DNA-binding transcription factor activity, and positive regulation of cytokine production (**Figures 3A, B**). Moreover, the results of KEGG pathway analysis showed that differential genes were enriched in the PI3K-Akt signaling pathway, Cytokine-cytokine receptor interaction, Human T -cell leukemia virus 1 infection, MAPK signaling pathway, and JAK-STAT signaling pathway (**Figures 3C, D**).

**Figure 4:**
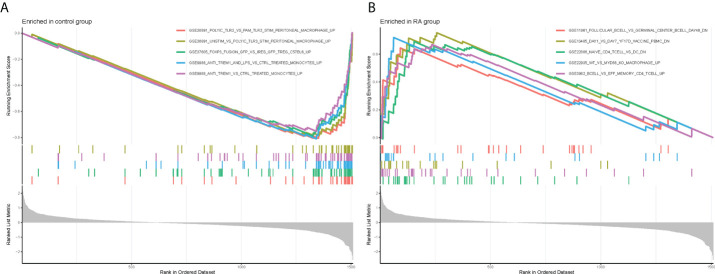
Gene Set Enrichment Analysis plots. The five significant enriched gene sets in the control group **(A)** and RA group **(B)**.

Meanwhile, to explore the possible mechanisms of immune function in RA progression, the enrichment of gene sets in the normal and RA groups was compared. The results of GSEA showed that gene sets GSE11961 follicular B cell vs. germinal center B cell day40 down, GSE13485 day1 vs. day7 YF17D vaccine PMBC down, GSE22886 naïve CD4 T cell vs. DC down, GSE22935 WT vs. MyD88 ko macrophage up, and GSE3982 B cell vs. eff memory CD4 T cell up were enriched in the RA group; while the gene sets GSE36891 polyic TLR3 vs. pam TLR2 stim peritoneal macrophage up, GSE36891 unstim vs. polyic TLR3 stim peritoneal macrophage up, GSE37605 FOXP3 fusion GFP vs. ires GFP Treg C57BL6 up, GSE9988 anti TREM1 and LPS vs. ctrl treated monocytes up, and GSE9988 anti TREM1 vs. ctrl treated monocytes up were active in the control group (**Figures 4A, B**). Overall, these results demonstrated that CRGs may play an important role in the pathogenesis and progression of RA.

### Identification of hub CRGs in RA

**Figure 5:**
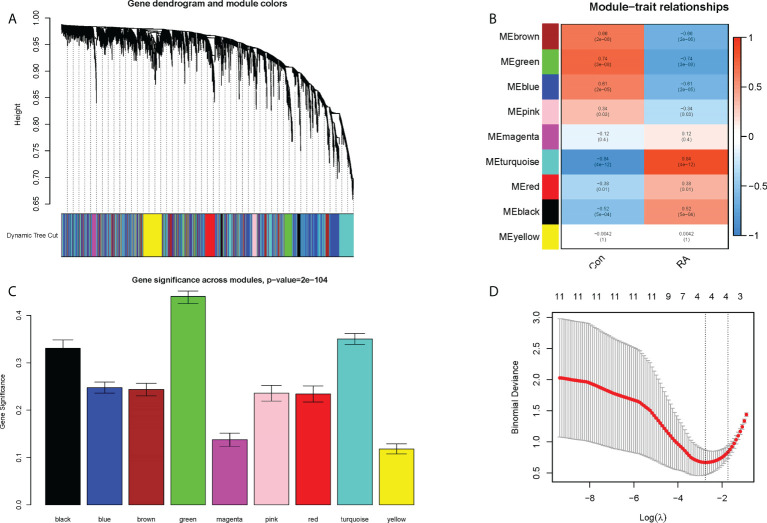
Screening of hub CRGs. **(A)** The cluster dendrogram of the genes. Each branch in the figure represented one gene, and every color below represented one co-expression module. **(B)** Heatmap of the module-trait relationships. **(C)** Distribution of average gene significance in the modules associated with the RA. **(D)** LASSO regression analysis.

Nine co-expression modules were constructed with the help of WGCNA, which were shown in different colors. The clustering dendrogram aggregated genes with common gene expression patterns in the same color modules (**Figure 5A**). The graph of module-trait correlation showed that turquoise, red and black modules were positively correlated with RA (MEturquoise: r = 0.84, P = 4e-12; MEred: r = 0.38, P = 0.01; MEblack: r = 0.52, P = 5e-04). In contrast, brown, green, blue and pink modules were negatively correlated with RA (MEbrown: r = -0.06, P = 2e-06; MEgreen: r = -0.74, P = 3e-08; MEblue: r = -0.61, P = 2e- 05; MEpink: r = -0.34, P = 0.03) (**Figure 5B**). Meanwhile, the green module including 29 candidate hub genes was identified as the hub module because of its highest gene significance (**Figure 5C**). Next, 23 CRGs associated with RA were obtained by taking the intersection of 490 DECRGs with 29 candidate hub genes in the green module (**Supplementary Table 3**). Finally, four hub CRGs were obtained by LASSO regression analysis, namely EGR1, FOSL2, GADD45B, and NFIL3 (**Figure 5D**).

### Immune cell infiltration and its correlation with hub CRGs

**Figure 6:**
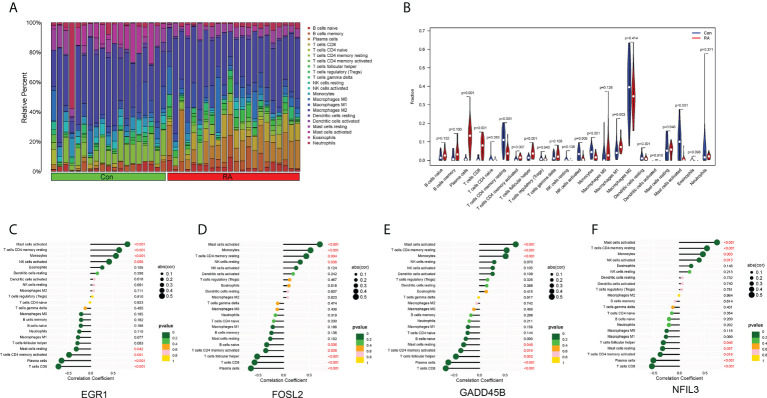
Immune cell infiltration. **(A)** Histogram showed the composition of 22 immune cells in each sample. **(B)** Violin plot showed the differences in infiltration levels of immune cells between RA and control groups. **(C–F)** Lollipop plots of the correlation between four hub CRGs and immune cells.

The landscape of immune cell infiltration in RA is essential for understanding the disease status. The content of 22 immune cells in the samples was calculated using the ‘CIBERSORT’ software, and the differences in the composition of immune cell subtypes between control and RA samples were observed (**Figures 6A, B**). It was evident that Plasma cells, CD8+ T cells, activated memory CD4+ T cells, T follicular helper (Tfh) cells, and M1 macrophages were significantly infiltrated in RA synovial tissue compared to normal control synovial tissue (**Figure 6B**).

The correlation between the four hub CRGs and immune cells was further analyzed. The results showed that these four hub CRGs were all positively correlated with activated Mast cells, resting memory CD4+ T cells, and Monocytes (**Figures 6C–F**). In contrast, all four hub CRGs were negatively correlated with Plasma cells, CD8+ T cells, and activated memory CD4+ T cells. In addition, FOSL2 and GADD45B were negatively correlated with Tfh cells.

### Diagnostic value of hub CRGs and nomogram

**Figure 7:**
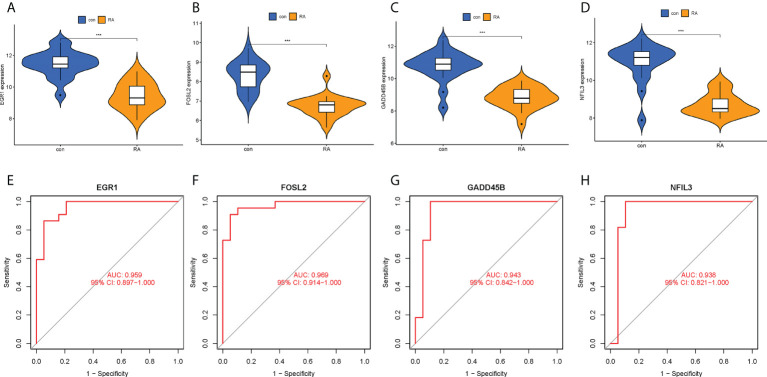
Expression levels and ROC curves of hub CRGs. **(A–D)** Violin plots of the difference in expression levels of hub CRGs between RA and control groups. **(E–H)** ROC curves manifested the diagnostic efficacy of each hub gene. ***p < 0.001. AUC, area under the curve.

The expression levels of the four hub CRGs were analyzed in the RA group versus the control group, which revealed that all hub CRGs were significantly downregulated in the RA group (**Figures 7A–D**). To calibrate the efficacy of four hub CRGs for RA diagnosis, the area under the ROC curve (AUC) was calculated for each gene. As shown in **Figures 7E–H**, the AUC values of EGR1, FOSL2, GADD45B, and NFIL3 were 0.959, 0.969, 0.943, and 0.938, respectively. The AUC values > 0.85 in all four hub genes indicated that these genes had excellent specificity and sensitivity for the diagnosis of RA.

**Figure 8:**
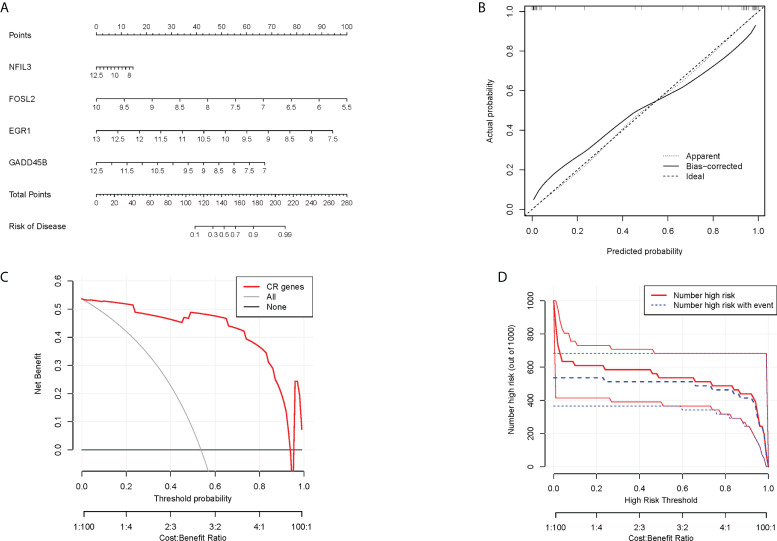
Nomogram model. **(A)** Nomogram of hub CRGs. **(B)** Calibration curve of the model. **(C)** Clinical decision curve. **(D)** Clinical impact curve (The red curve indicated the number of people classified as positive (high risk) by the model at each threshold probability; the blue curve indicated the number of true positive people at each threshold probability).

In order to obtain a more clinically applicable diagnostic model, a nomogram of four hub CRGs was constructed (**Figure 8A**). In the nomogram, the risk of RA disease in patients can be calculated based on the scoring of each gene expression. Of course, the reliability of the nomogram was also verified by the decision curve and clinical impact curve analysis, which showed that this model has a high diagnostic value (**Figures 8B–D**).

### Validation of hub CRGs

**Figure 9:**
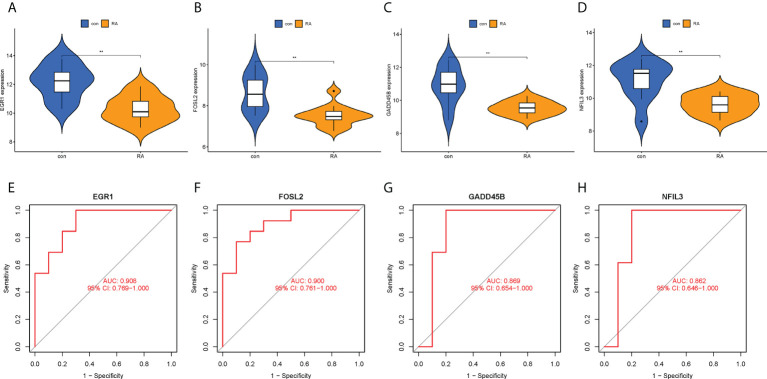
Validation of hub CRGs with GSE55457 dataset. **(A–D)** Violin plots of the differences in expression levels of four hub genes between RA and control groups. **(E–H)** ROC curves of diagnostic efficacy of each hub gene for RA. **p < 0.01. AUC, area under the curve.

To confirm the clinical utility of the screened hub CRGs, their diagnostic value and expression levels were validated in another external dataset GSE55457. It was observed that EGR1, FOSL2, GADD45B, and NFIL3 showed low expression in the RA group, which was consistent with our previous findings (**Figures 9A–D**). Meanwhile, the AUCs of the four hub CRGs were calculated, with 0.908 for EGR1, 0.900 for FOSL2, 0.869 for GADD45B, and 0.862 for NFIL3 (**Figures 9E–H**). Therefore, EGR1, FOSL2, GADD45B, and NFIL3 might be used as the diagnostic biomarkers for RA.

## Discussion

Rheumatoid arthritis is a systemic autoimmune disease characterized by synovitis. Patients with RA have typical clinical symptoms of circadian rhythm variability (6, 7). Circadian rhythm is a natural endogenous process and its physiological function is controlled by a set of CRGs. Disturbances of CRGs have detrimental effects on both innate and acquired immune function, which significantly enhances pro-inflammatory responses and susceptibility to autoimmune diseases (21). To date, many CRGs have been found to be involved in the regulation of inflammatory pathways and immune cell differentiation in autoimmune diseases (22, 23). Conversely, autoimmune and inflammatory diseases can also directly affect the expression of CRGs, which may lead to a vicious cycle of inflammation (24). In this study, we identified circadian rhythm-associated hub biomarkers in RA by bioinformatics methods, analyzed their association with immune cells, and initially explored their potential molecular mechanisms in the pathogenesis and development of RA.

Identification of disease-related modules by WGCNA to explore functional pathways and candidate biomarkers has been shown to be an effective approach (20, 25). In this study, the genes highly associated with RA extracted by WGCNA were intersected with previous DECRGs to obtain intersecting genes with both differences and correlations (**Figures 5A–C**). Then, the following four hub CRGs were finally obtained by LASSO regression analysis, namely EGR1, FOSL2, GADD45B, and NFIL3 (**Figure 5D**). These four hub genes were significantly differentially expressed in normal synovial tissues and RA synovial tissues, and had excellent diagnostic efficacy for RA (**Figures 7E–H**). In RA synovial tissues, EGR1, FOSL2, GADD45B, and NFIL3 were significantly downregulated (**Figures 7A–D**). Finally, further validation for these genes was performed by an external dataset (GSE89408), which made our results more convincing (**Figure 9**).

The nuclear factor interleukin 3 (NFIL3) gene encodes a protein acting as a transcriptional regulator. It has been suggested that NFIL3 protein repressed circadian expression of PER1 and PER2 and therefore played a negative regulatory role in circadian rhythm (26). NFIL3 can enhance cancer-associated inflammation mediated by the NF-κB signaling pathway in breast cancer patients (27). Early growth response protein 1 (EGR1) is a transcription factor that can be rapidly induced by growth factors, cytokines, and stress signals, such as radiation, injury, or mechanical stress. It has been found that EGR1 can regulate the amplitude of CRGs (such as ARNTL/BMAL1, PER2, and NR1D1) expression by activating PER1 transcription in the liver (28, 29). Meanwhile, EGR1 can regulate the rhythmic expression of the nuclear clock gene ARNTL/BMAL1 in the suprachiasmatic nucleus of the hypothalamus (30). Previous studies have found that the core clock genes CLOCK, BMAL1, and PER were also expressed in both synovial tissue and fibroblasts in RA, while knockout of these genes produced an inflammatory response (31). It has been reported that circadian rhythmicity of PER2 and PER3 expression was lost in CD14+ monocytes of RA patients and that the amplitude of BMAL1 expression was lower than in healthy ones (32). The above studies suggested that NFIL3 and EGR1 might be closely associated with circadian rhythms in RA. However, the relationships between RA and FOSL2 and GADD45B are still unclear and need to be further investigated.

Innate and acquired immune responses play an integral role in the pathogenesis of RA. The complex interaction and activation of infiltrating immune cells were key factors in the development of synovitis and persistent joint damage, as reported in mounting studies (19, 33). Zhou et al. identified 10 immune cells with differential infiltration levels and 202 differentially expressed genes between synovial samples from RA and normal patients and obtained five hub genes that could be used as diagnostic biomarkers for RA by bioinformatics analysis (19). They also indicated that GZMA-Tfh cells, CCL5-M1 macrophages, and CXCR4-memory activated CD4+ T cells/Tfh cells may participate in the occurrence and development of RA. Moreover, several studies have obtained other different diagnostic biomarkers for RA and reported that these biomarkers were closely associated with some immune cells, such as monocytes, CD8+ T cells, Tfh cells, and mast cells (33, 34).

In this study, the hub CRGs were also found to play a crucial role in RA-related immune responses (**Figures 6C–F**). It has been noted that CRGs are commonly expressed in most immune cells and exhibit circadian oscillations with fixed rhythms, as well as that CRGs play important roles in a wide range of immune regulatory processes, including phagocytosis, apoptosis, synthesis, and release of cytokines, chemokines and cytolytic factors (15, 16, 35). For example, the circadian gene BMAL1 could regulate the circadian oscillations of LY6C(hi) inflammatory monocytes (36). Studies have shown that NFIL3 downregulation can impair the function of Th2 and Treg cells (37, 38). Meanwhile, NFIL3 could protect pro-B cells from programmed cell death and be required for the development of NK cells (39). FOSL2 can promote systemic autoimmune and inflammatory responses by inhibiting Treg cell development (40). EGR1 could attenuate the activity of macrophage inflammatory enhancers (41). Therefore, it can be hypothesized that circadian changes due to differential expression of hub CRGs in RA may lead to disturbed immune responses, which may be caused by CRGs mutations, environmental disruptions, and RA itself. However, to our knowledge, the dominant cells that drive cytokine circadian oscillations and pathology in RA are still unknown. Further experimental studies are required to investigate the specific role of hub CRGs identified in this study in the circadian regulation of RA.

The sleep-wake cycle is one of the most important endogenous biological rhythms and is closely associated with inflammatory and autoimmune diseases (42). It has been demonstrated that disruption of circadian rhythms in staff with work shifts, such as night shifts, overtime, rotations, and frequent travel, due to misaligned sleep and wake schedules, could lead to immune system disorders eventually (42–44). Notably, the onset and progression of RA is also influenced by other environmental factors, such as GCs, sex hormones, and vitamin D (45, 46). GCs are the most important anti-inflammatory substances and are associated with circadian variations of cortisol secretion (42). The symptoms of morning stiffness in RA are associated with increased pro-inflammatory cytokines and deficiency of GCs at night and in the morning (47). Therefore, patients with RA may benefit from the use of GCs at late night, as they can counteract the peak of inflammatory cytokines (48). In addition, during active RA, gonadal and adrenal androgens are significantly reduced in inflamed synovial tissues/fluids as a result of the inflammatory reaction (49). Androgens show anti-inflammatory effects *in vitro* and *in vivo* and can reduce the secretion of cytokines such as TNF, IL-1β, and IL-6 by monocytes and macrophages, and IL-33 by mast cells (50). Hence, the treatment with androgens as hormone replacement therapy may be promising for men with RA (45). Furthermore, decreased serum levels of vitamin D and its metabolites in RA may increase the risk of fibroblast-like synovial cell-mediated invasion and erosion of cartilage as well as bone (51). Vitamin D could downregulate pro-inflammatory cytokines and promote the action of anti-inflammatory cytokines through multiple immune cells and pathways (46, 52). Considering the above, the epigenetic impact of these environmental factors needs to be considered in future studies of hub CRGs and related cells.

This study also has some limitations. Firstly, this study was performed only at the gene transcription level, and the results may not be applicable to studies at the protein level. Secondly, the results of this study were not validated by further *in vivo* and *in vitro* experiments. However, we have performed validation in an external dataset to ensure the accuracy of the results. Finally, a follow-up comparison of circadian gene expression between RA and osteoarthritis is necessary because they may have similar pathological processes.

## Conclusion

In summary, with the help of WGCNA and LASSO analysis, one hub module (green module) and four hub genes (EGR1, FOSL2, GADD45B, and NFIL3) associated with the circadian rhythm in RA were screened in this study. These hub CRGs have excellent diagnostic value for RA and may be involved in the pathological process of RA by disrupting the rhythmic oscillations of cytokines through immune-related pathways. This study provided new perspectives on the diagnosis, immune infiltration pattern, and circadian rhythm mechanism in RA. However, further prospective experiments are needed to confirm the findings of the present study.

## Data availability statement

The original contributions presented in the study are included in the article/**Supplementary Material**. Further inquiries can be directed to the corresponding authors.

## Author contributions

PW: writing-original draft. PW, JW, and YZ: conceptualization, project administration, and writing-review and editing. PW, TM, and BZ: data curation and methodology. LH, YW, and JG: formal analysis and validation. PW, TM, and WS: visualization and software. All authors contributed to the article and approved the submitted version.

## Funding

This work was supported by the Youth Cultivation Project of Xi’an Health Commission (Program No. 2020qn18) and the Key Research and Development Program of Shaanxi Province (Program No. 2022SF-237).

## Acknowledgments

We acknowledge the GEO database for providing their platforms as well as their contributors for uploading meaningful datasets.

## Conflict of interest

The authors declare that the research was conducted in the absence of any commercial or financial relationships that could be construed as a potential conflict of interest.

## Publisher’s note

All claims expressed in this article are solely those of the authors and do not necessarily represent those of their affiliated organizations, or those of the publisher, the editors and the reviewers. Any product that may be evaluated in this article, or claim that may be made by its manufacturer, is not guaranteed or endorsed by the publisher.
